# Complacency in Artificial Intelligence Models and Its Impact on Psychosis: A Media Discourse Analysis

**DOI:** 10.1192/j.eurpsy.2026.11349

**Published:** 2026-07-31

**Authors:** K. M. Munera-Luque, R. Pupo-Díaz, Z. Miranda-Sánchez

**Affiliations:** 1 Magdalena, Universidad Cooperativa de Colombia; 2 Magdalena, Universidad del Magdalena, Santa Marta, Colombia

## Abstract

**Introduction:**

Artificial intelligence (AI) systems increasingly shape social perceptions of reality and influence public discourses on mental health. Recent evidence suggests that AI models often display communicative complacency—a tendency to please users, avoid disagreement, and reinforce their statements. This phenomenon, particularly evident in conversational systems, has psychological and social implications, as it may contribute to the validation of cognitive distortions resembling those linked to psychotic processes. Media narratives play a key role in amplifying or moderating these effects, shaping public understanding of both AI and mental disorders.

**Objectives:**

This study explored how complacency attributed to AI models in journalistic discourse relates to concepts associated with psychosis, especially delusional validation and social feedback loops. It aimed to identify linguistic and symbolic parallels revealing psychosocial risks derived from media representations of complacent human–AI interactions.

**Methods:**

A qualitative and lexicographic content analysis was conducted on 80 press articles published between 2022 and 2025 in major international outlets. Texts were analyzed through thematic coding using ATLAS.ti software, focusing on patterns of complacency, validation, and anthropomorphism in AI descriptions. The resulting categories were compared with psychological constructs related to psychosis, such as external attribution, distorted feedback, and loss of agency.

**Results:**

Media discourse often portrays AI models through traits of communicative complacency, describing them as “empathetic,” “understanding,” or “harmless,” thereby reinforcing anthropomorphic projections and emotional attributions toward non-human agents. These narratives normalize affective reciprocity with automated systems, reproducing validation and reinforcement mechanisms akin to delusional dynamics. Lexicographic analysis showed that semantic fields of comfort, obedience, and submission were predominant, with a 62% co-occurrence between “useful” and “obedient.” Overall, findings reveal a convergence between the social construction of complacent AI and the phenomenology of psychotic validation, where the pursuit of confirmation replaces critical reality testing.

**Conclusions:**

Complacency within AI models, as reflected in media narratives, contributes to imaginaries that blur boundaries between subjective belief and confirmatory generative feedback. This may foster cognitive environments conducive to delusional validation, underscoring the need for interdisciplinary monitoring of human–AI interaction discourses. Future research should integrate psychological, linguistic, and computational perspectives to assess how algorithms optimized for user approval influence thought regulation and collective mental health.

**Disclosure of Interest:**

None Declared

